# *PARK2* as a susceptibility factor for nontuberculous mycobacterial pulmonary disease

**DOI:** 10.1186/s12931-024-02946-4

**Published:** 2024-08-14

**Authors:** Youngmok Park, Ji Won Hong, Eunsol Ahn, Heon Yung Gee, Young Ae Kang

**Affiliations:** 1 Division of Pulmonary and Critical Care Medicine, Department of Internal Medicine, Severance Hospital, Yonsei University College of Medicine, Seoul, Republic of Korea; 2 Institute for Innovation in Digital Healthcare, Yonsei University, Seoul, Republic of Korea; 3 Departments of Pharmacology, Graduate School of Medical Science, Brain Korea 21 Project, Yonsei University College of Medicine, Seoul, Republic of Korea; 4 Division of Vaccine Research, International Tuberculosis Research Center, Seoul, Republic of Korea; 5 Woo Choo Lee Institute for Precision Drug Development, Seoul, Republic of Korea; 6 Institute of Immunology and Immunological Diseases, Yonsei University College of Medicine, Seoul, Republic of Korea

**Keywords:** Autophagy, Nontuberculous mycobacteria, Gene expression profiling, Autophagy, Ubiquitination

## Abstract

**Background:**

The genetic signatures associated with the susceptibility to nontuberculous mycobacterial pulmonary disease (NTM-PD) are still unknown. In this study, we performed RNA sequencing to explore gene expression profiles and represent characteristic factor in NTM-PD.

**Methods:**

Peripheral blood samples were collected from patients with NTM-PD and healthy individuals (controls). Differentially expressed genes (DEGs) were identified by RNA sequencing and subjected to functional enrichment and immune cell deconvolution analyses.

**Results:**

We enrolled 48 participants, including 26 patients with NTM-PD (median age, 58.0 years; 84.6% female), and 22 healthy controls (median age, 58.5 years; 90.9% female). We identified 21 upregulated and 44 downregulated DEGs in the NTM-PD group compared to those in the control group. NTM infection did not have a significant impact on gene expression in the NTM-PD group compared to the control group, and there were no differences in the proportion of immune cells. However, through gene ontology (GO), gene set enrichment analysis (GSEA), and protein-protein interaction (PPI) analysis, we discovered that *PARK2* is a key factor associated with NTM-PD. The *PARK2* gene, which is linked to the ubiquitination pathway, was downregulated in the NTM-PD group (fold change, − 1.314, *P* = 0.047). The expression levels of *PARK2* remained unaltered after favorable treatment outcomes, suggesting that the gene is associated with host susceptibility rather than with the outcomes of infection or inflammation. The area under the receiver operating characteristic curve for the *PARK2* gene diagnosing NTM-PD was 0.813 (95% confidence interval, 0.694–0.932).

**Conclusion:**

We identified the genetic signatures associated with NTM-PD in a cohort of Korean patients. The *PARK2* gene presents as a potential susceptibility factor in NTM-PD .

**Supplementary Information:**

The online version contains supplementary material available at 10.1186/s12931-024-02946-4.

## Introduction

Nontuberculous mycobacteria (NTM) are ubiquitous environmental organisms found in natural and drinking water systems, pools, hot tubs, and soil [1]. NTM can infect various tissues and body fluids and most commonly cause pulmonary disease (PD) [2]. The worldwide incidence and prevalence of NTM-PD are increasing, affecting both immunocompromised and immuno-competent patients [3, 4]. Furthermore, the distribution of NTM varies depending on the geographic location, primarily due to environmental factors [5]. Consequently, the characteristics of species distribution in NTM-PD patients differ across different regions [6]. 

NTM-PD presents numerous challenges for physicians. The diagnosis of NTM-PD is complex, requiring repeated culture results for certain NTM species and radiographic correlation of chest images and related symptoms [2, 7]. The clinical course of NTM-PD is diverse and unpredictable; some cases progress rapidly, whereas others remain stable without treatment or experience spontaneous remission [8]. The factors that determine treatment response are not fully understood, and based on current knowledge, the timings of treatment initiation need further investigation [9]. Consequently, the treatment outcomes are often disappointing. Treatment success rates for *Mycobacterium avium* complex PD range from 55 to 65% [10], while those for *Mycobacterium abscessus* PD range from 24 to 46%, deeming the condition incurable [11]. Moreover, the current treatment regimens are suboptimal due to the side effects they cause [12]. Therefore, in terms of unsatisfying treatment outcomes and drug-related toxicity, current regimens are not optimal.

To reduce diagnostic barriers and monitor treatment response, potential biomarkers of NTM-PD such as interleukin 17, carbohydrate antigen 19 − 9, anti-glycopeptidolipid IgA, and anti-interferon (IFN)-γ autoantibody titer have been identified [13, 14]. 

We hypothesized that there would be differences in the blood gene expression profiles between patients with NTM-PD and healthy individuals (controls). Therefore, this study aimed to explore the genetic characteristics of NTM-PD and identify specific factor through RNA sequencing.

## Methods

### Study participants and blood sample collection

Peripheral blood samples were collected from patients with NTM-PD and healthy controls between 2015 and 2019. We collected 2.5 mL of peripheral blood into the PAXgene RNA tube (Becton Dickinson and Co., Franklin Lakes, NJ, USA) and stored it at − 80 ℃ until analysis. For patients with NTM-PD, blood samples were collected before and at the end of treatment. Individuals with malignancies, end-stage renal disease, or human immunodeficiency virus infection were excluded from the study. Healthy controls were defined as individuals without respiratory symptoms, chest radiographic abnormalities, or a medical history of chronic lung diseases.

### Clinical data of study participants

Clinical data of the participants were collected from their electronic health records. Medical charts were reviewed, considering factors such as age, sex, body mass index, and underlying diseases. Patients with NTM-PD were diagnosed according to guidelines 2, 7. Upon reviewing the chest computed tomography results, the radiological types were classified as nodular bronchiectatic or fibrocavitary. Disease severity was determined based on the extent of lung involvement and sputum acid-fast bacilli smear results. Treatment outcomes were defined using the NTM-NET consensus statement [15]. 

### RNA sequencing

RNA was extracted from peripheral blood samples of patients with NTM-PD using the PAXgene Blood RNA Kit (PreAnalytiX, Hombrechtikon, Switzerland) according to manufacturer’s instructions. The integrity of total RNA was evaluated using the Agilent 2100 Bioanalyzer (Agilent Technologies, Santa Clara, CA, USA) which assigns it an RNA Integrity Number (RIN). RNA with an optical density of 260/280 ≥ 1.8 and RIN ≥ 7 were selected for the subsequent experiments. The total RNA sequencing libraries were prepared according to the manufacturer’s instructions (Illumina TruSeq Stranded Total RNA Sample Prep Kit with Ribo-Zero Globin, Illumina, San Diego, CA, USA). The process involved removing ribosomal RNA from 500 ng of total RNA using Ribo-Zero Globin reagent, utilizing biotinylated probes to bind rRNA species selectively. After purification, the rRNA-depleted total RNA was fragmented into small pieces using divalent cations at an elevated temperature. The resulting cleaved RNA fragments were converted into first-strand cDNA using reverse transcriptase and random primers. Subsequently, second-strand cDNA was synthesized using DNA Polymerase I and RNase H. These cDNA fragments were modified with a single ‘A’ base and ligated with an adapter. The products were purified and enriched through PCR to create the final cDNA library.

The quality of the amplified libraries was confirmed through capillary electrophoresis using Bioanalyzer (Agilent Technologies). After performing real-time polymerase chain reaction with SYBR Green PCR Master Mix (Applied Biosystems, Foster City, CA, USA), we combined the libraries that were index tagged in equimolar amounts into a pool. Finally, RNA sequencing was performed using a NovaSeq 6000 system (Illumina) following the provided protocols for 2 × 100 sequencing. The reads for each sample were mapped to the reference genome of the Human Genome Reference Consortium Human Build 37 (GRCh37, hg19) [16] using CLC Genomics Workbench 9.5.3 software (Qiagen). We generated gene expression values in the normalized form of TPM (Transcripts Per Million) values.

### Bioinformatics analysis

All differentially expressed genes (DEGs) were chosen using FDR < 0.05. We visualised the RNA-sequencing analysis including hierarchical clustering heatmaps and principal component analysis (PCA) using R studio v3.6.3. Functional enrichment with Gene ontology was performed using Database for Annotation, Visualization, and Integrated Discovery (DAVID) functional annotation tool and Gene Set Enrichment Analysis (GSEA) v4.1.0 was performed using Hallmark gene sets from the Molecular Signatures Database (MSigDB).

Using the STRING (v1.7.0, https://string-db.org) [17] database, the protein-protein connections were assessed among the DEGs, and protein interactions were plotted using Cytoscape (v3.8, https://cytoscape.org). We identified human genes with relevant functional gene ontology (GO). For integrative analysis, we employed both the Database for Annotation, Visualization, and Integrated Discovery (DAVID) functional annotation tool and the ClueGO (v2.5.8)/CluePedia (v1.5.8) plugin of Cytoscape to complementarily identify the DEGs involved in the GO terms and pathways. ClueGO combines GO terms and pathways from Kyoto Encyclopedia of Genes (KEGG), Reactome, and Wiki, providing a structured GO term or pathway network from the DEG dataset [18]. In addition, the CluePedia integrates into the ClueGO network of terms/pathways, linking genes based on in silico and experimental information [19]. To determine significance, we applied a threshold of *P* values < 0.05 for the study of molecular/biological/cellular function GO and enrichment of pathway analysis for DEGs.

### Immune cell deconvolution

To analyze the composition of immune cells in our samples, we employed the CIBERSORTx platform, a computational method designed to characterize the cell composition of complex tissues based on their gene expression profiles [20]. Analysis was performed using the LM22 signature matrix, which consists of 22 distinct immune cell subtypes, including B cells, T cells, natural killer cells, macrophages, dendritic cells, and myeloid subsets [21]. The analysis was conducted in the absolute mode, which enabled a more quantitative interpretation of the results by providing the exact proportions of each cell type.

### Statistical analyses

The median values of the variables between the two groups were compared using the Mann–Whitney *U* test. Categorical variables were compared using Fisher’s exact test. Wilcoxon signed-rank test was conducted to compare the median gene expression values between pre- and post-treatment samples.

Receiver operating characteristic curves were generated to assess the clinical relevance of the identified markers and the area under the curve (AUC) was calculated to determine the optimal cutoff value and discriminatory capacity. Sensitivity, specificity, positive predictive value, and negative predictive value were evaluated based on the optimal cutoff values. Differences with a two-sided *P* value below 0.05 were considered statistically significant.

## Results

### Distinct transcriptional responses are induced in patients with NTM-PD

**Fig. 1:**
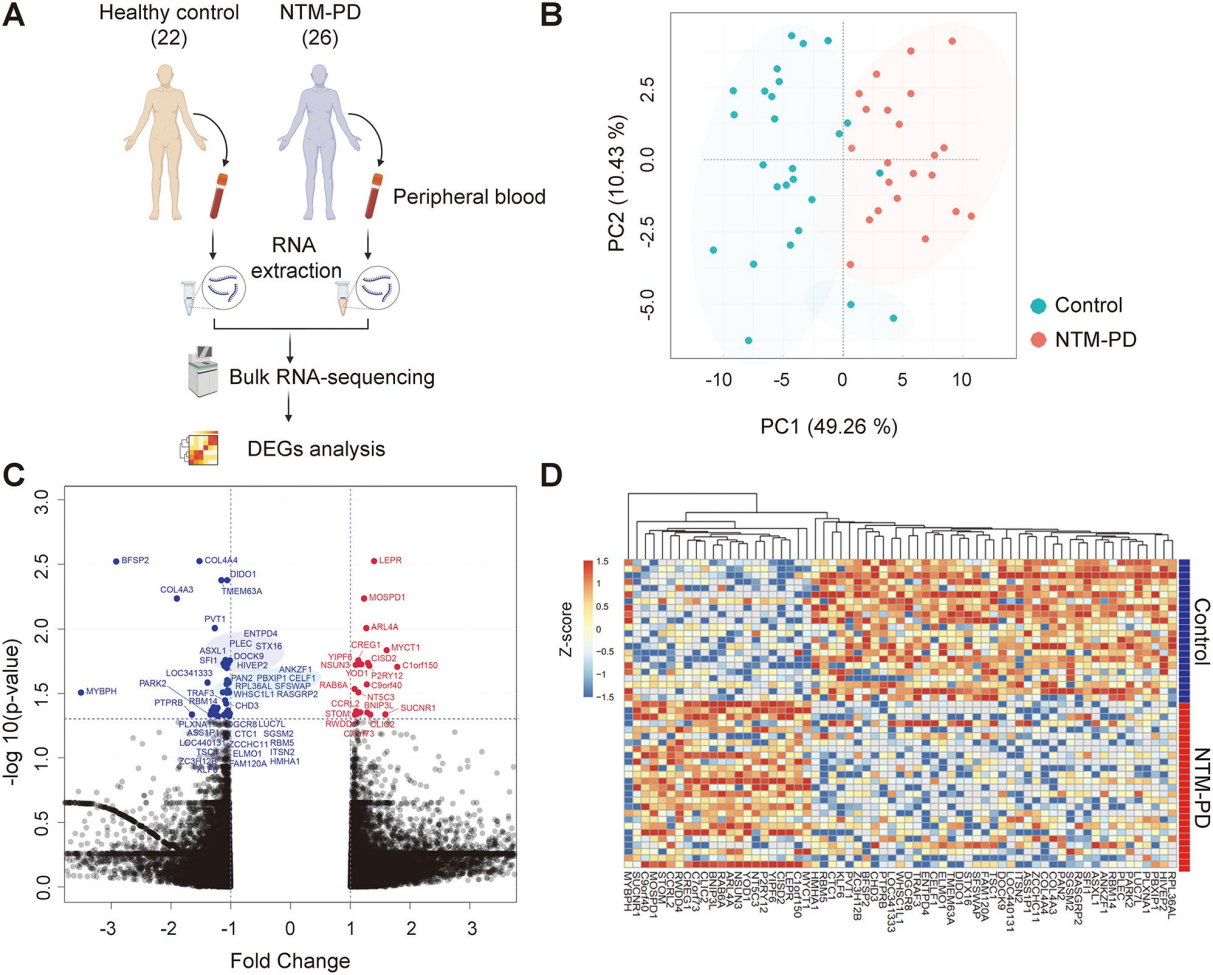
Transcriptomic analysis in healthy control and NTM-PD patient group. (**A**) Schematic diagram of bulk RNA-sequencing. (**B**) Principal component analysis (PCA) of genes corresponding to Differentially Expressed Genes (DEGs). (**C**) Volcano plot visualizing the DEGs. The vertical dotted lines are positioned at fold change of 1 or -1. The horizontal dotted lines are positioned at false discovery rate (FDR) = 0.05. (**D**) Heatmap generated using 65 DEGs between the case and control groups. The expression levels of the DEGs were converted to a heatmap, with red representing upregulation and blue representing downregulation

Bulk RNA sequencing was performed using blood samples from 22 healthy controls and 26 patients with NTM-PD (Table 1; Fig. 1A). Supplementary Fig. 1 illustrates the results of the unsupervised principal component analysis (PCA) conducted on the NTM-PD and control groups. However, these components did not effectively differentiate between the two groups. We conducted DEGs analysis using a false discovery rate (FDR) < 0.05, and PCA using these DEGs effectively distinguished between the two groups (Fig. 1B). Compared to the control group, 21 upregulated and 44 downregulated genes were identified, which are depicted in a volcano plot (Fig. 1C). A heatmap representing the expression levels of these DEGs is shown in Fig. 1D. Supplementary Tables 1 and 2 present the lists of upregulated and downregulated genes in the case group compared with those in the control group.

**Table 1:** Baseline characteristics of the study population

	NTM-PD (*N* = 26)	Healthy controls (*N* = 22)	*P*-value
Age, years	58.0 [48.0–64.0]	58.5 [56.0–60.0]	0.967
Sex, female	22 (84.6)	20 (90.9)	0.827
Height, cm	159.8 [157.0–162.0]	157.0 [155.0–166.0]	0.101
Weight, kg	52.0 [49.6–57.0]	58.0 [54.0–63.0]	0.021
BMI, kg/m^2^	20.9 [19.8–22.0]	23.5 [21.8–24.4]	0.002
BMI < 18.5 kg/m^2^	4 (15.4)	0 (0.0)	0.162
Ever smoker	3 (11.5)	0 (0.0)	0.295
Comorbidity			
Hypertension	4 (15.4)	5 (22.7)	0.781
Diabetes mellitus	1 (3.8)	1 (4.5)	> 0.999
Bronchiectasis	24 (92.3)		
COPD	5 (19.2)		
History of tuberculosis	7 (26.9)		
Previous NTM treatment	3 (11.5)		
Causative organism			
* M. avium* complex	20 (76.9)		
* M. abscessus*	4 (15.4)		
Others	2 (7.7)		
Radiologic findings			
Non-cavitary NB	13 (50.0)		
Cavitary NB	10 (38.5)		
Fibrocavitary	3 (11.5)		
Extent, ≥ 3 lobes	21 (80.8)		
Sputum smear positivity	7 (26.9)		
Presence of cavity	13 (50.0)		

*Note* Data are presented as median [interquartile range] or number (percentage) Abbreviations: BMI, body mass index; COPD chronic obstructive pulmonary disease; NB nodular bronehiectatic form; NTM-PD nontuberculous mycobacterial pulmonary disease

### Functional enrichment analysis in NTM-PD

**Fig. 2:**
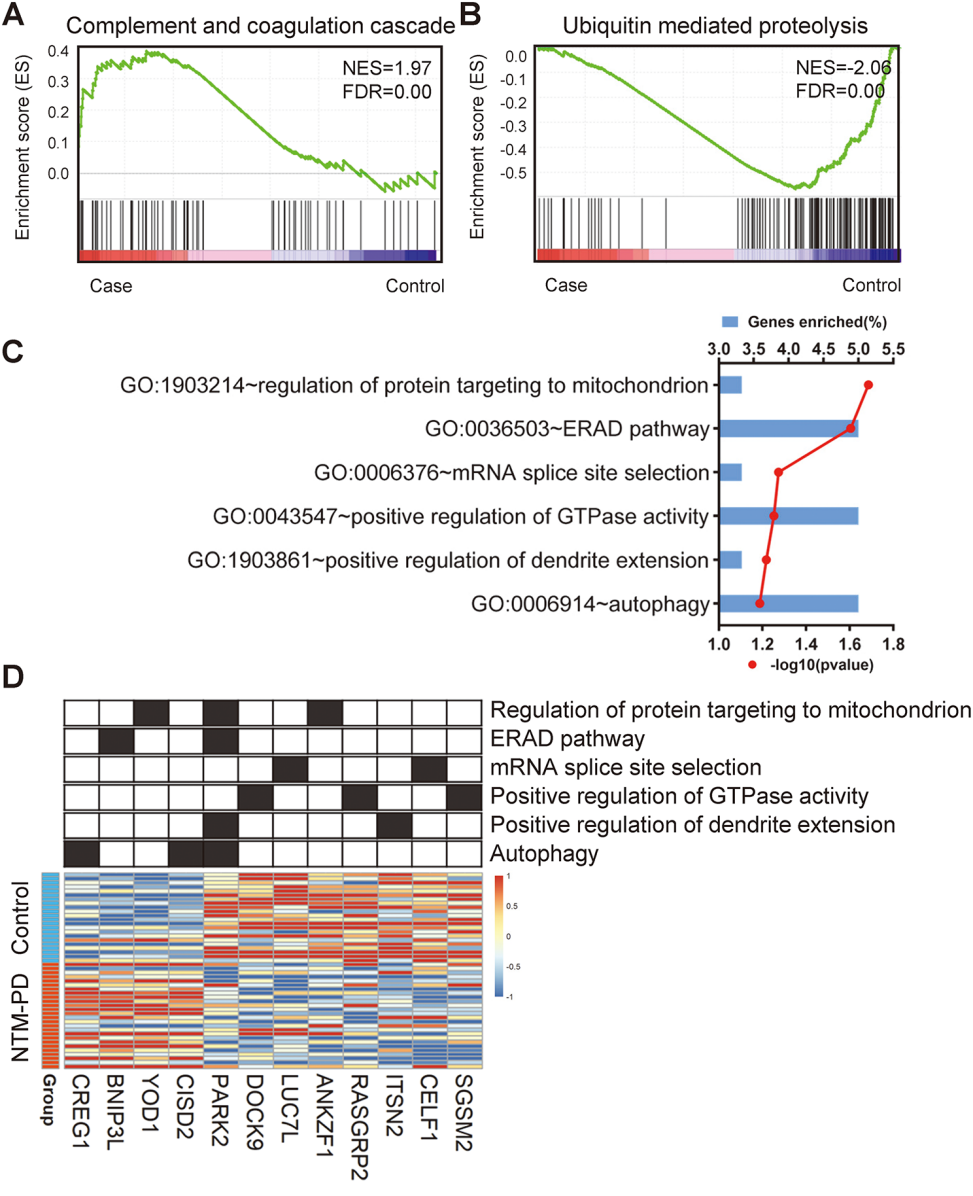
GSEA and GO analysis of Differentially Expressed Genes (DEGs). Gene set enrichment analysis (GSEA) of KEGG pathways of NTM-PD group compared to the control group. The enrichment plots for the complement and coagulation cascade (**A**) and ubiquitin-mediated proteolysis pathway (**B**) are shown. NES: normalized enrichment score. (**C**) GO analysis of biological process using DEGs. The bars represent the gene enrichment ratio for each term, and the red lines indicate -log10 (*P*-values). (**D**) Heatmap of genes belonging to biological process terms among the DEGs. Red indicates upregulation and blue indicates downregulation. The grid above the heatmap indicates gene assignments for GO terms

Next, we conducted Gene Set Enrichment Analysis (GSEA) related to Kyoto Encyclopedia of Genes and Genomes (KEGG) pathways and observed significant upregulation of genes associated with the complement and coagulation cascade in the NTM-PD group compared with the control group (Fig. 2A). The gene set associated with the ubiquitin-mediated proteolysis pathway was downregulated in the NTM-PD group compared with that in the control group (Fig. 2B). We analyzed the Gene Ontology (GO) of the DEGs between the NTM-PD and control groups. GO analysis of DEGs revealed enrichment of biological processes related to the endoplasmic reticulum-associated protein degradation (ERAD) pathway, regulation of protein targeting to mitochondria, and autophagy (Fig. 2C). We identified that 12 genes among the DEGs are involved in each biological process (Fig. 2D). The molecular function of DEGs was primarily associated with protein binding, and they were distributed in the cytosol, nucleus, and membrane (Supplementary Fig. 2).

### Protein-protein interaction analyses in NTM-PD

**Fig. 3:**
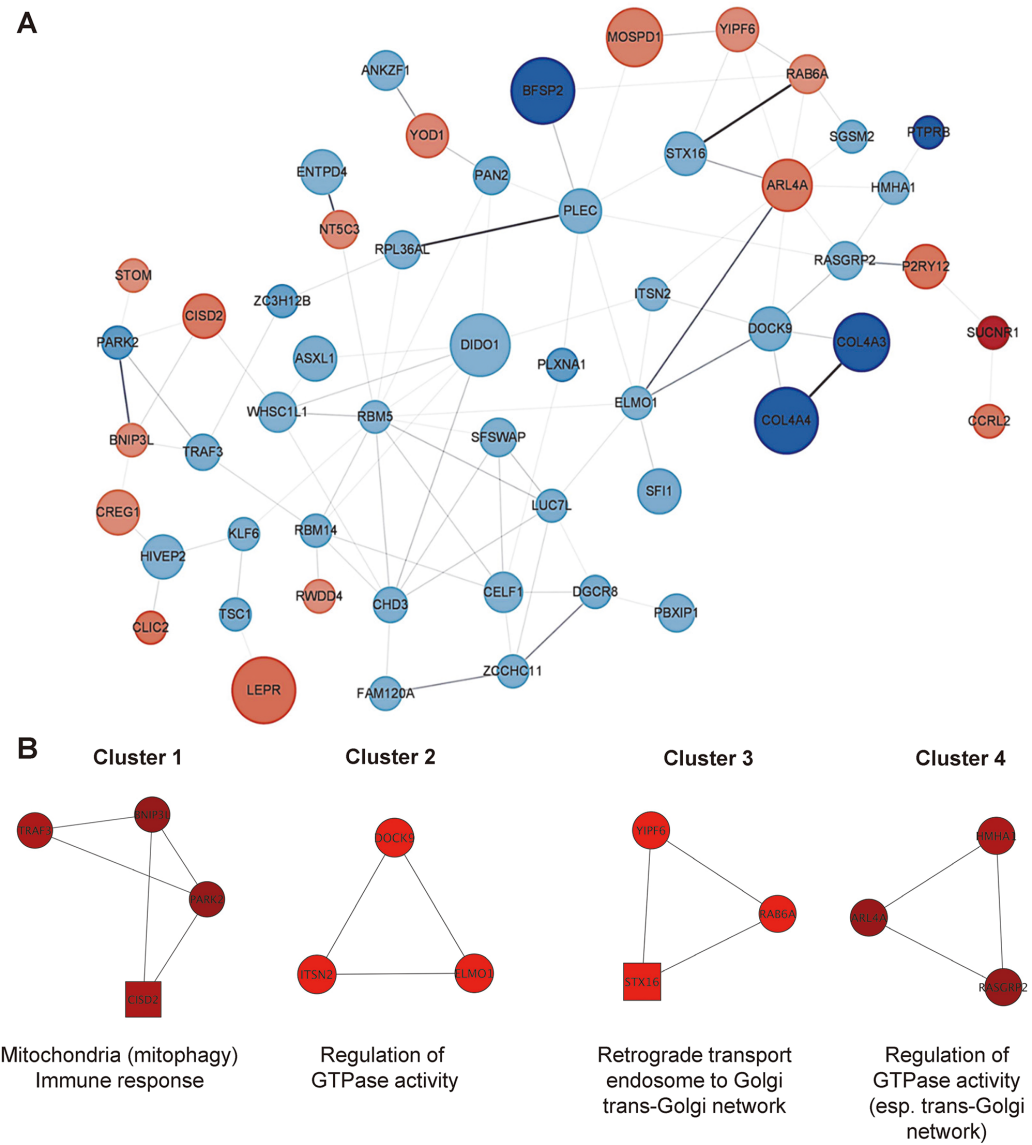
Protein-protein interaction analysis of NTM-PD. (**A**) Co-expression protein network construction. Red nodes represent significantly upregulated genes; blue nodes represent significantly downregulated genes. The nodes denote the number of proteins, while the edges represent their interactions. Node size is inversely related to the *P* value; edge color and edge width are directly related to the confidence score. (**B**) Modules of protein-protein interaction networks. The molecular complex detection (MCODE) plugin of Cytoscape was used for the analysis. The cluster finding parameters included a degree cutoff of 2 to exclude loops, a node score cutoff of 0.2, a kappa score of 2, and a max depth of 100, which limits the cluster size for co-expressing networks. Node shapes indicate the cluster status of the nodes. A square represents the seed (highest scoring rnode in the cluster), and a circle represents clustered proteins. Node color represents the node score; a range from black to red indicates the MCODE computed node scores (lowest to highest, respectively)

The protein-protein interactions among the DEGs were assessed and plotted, as shown in Fig. 3. A confidence level of 0.15 was set as the minimum requirement for the interaction scores. Figure 3A shows the protein-protein interaction network. The molecular complex detection (MCODE) plugin of Cytoscape was used to interpret closely interlinked regions in clusters from a network of proteins. Four clusters were identified that were related to immune response, intracellular transportation, and GTPase regulation (Fig. 3B). Among the four PPI clusters, cluster 1, corresponding to immune response, exhibited a higher interaction score compared to the other clusters (Supplementary Table 3).

Additionally, we conducted an immune cell deconvolution analysis of the NTM-PD and control groups. The differential distribution of immune cells between the NTM-PD and control groups was visualized using box plots in Supplementary Fig. 3. Neutrophils were the most common cell type in both groups (NTM-PD, 7.0% [5.4–9.2%]; controls, 6.6% [5.5–8.7%]; *P* = 0.644), followed by monocytes (NTM-PD, 3.3% [2.9–3.8%]; controls, 3.4% [3.1–3.9%]; *P* = 0.601). However, infection did not result in changes in the proportion of immune cell types.

### *PARK2* gene as a susceptibility factor for NTM-PD

Based on the previous results, we explored factors that are characteristic of the NTM-PD group compared to the control group. As a result, we focused on the *PARK2* gene, and the reasons for this are as follows: 1). In the biological process categories classified through GO analysis, *PARK2* is associated with most biological processes. 2). Among the genes related to the mitochondria (mitophagy) immune response cluster confirmed by PPI analysis, *PARK2* showed significant changes in expression between the control group and NTM-PD group. 3). In GSEA analysis, the pathway with a high NES score was ubiquitin-mediated proteolysis, and *PARK2* is known to be a gene associated with the ubiquitination pathway.

**Fig. 4:**
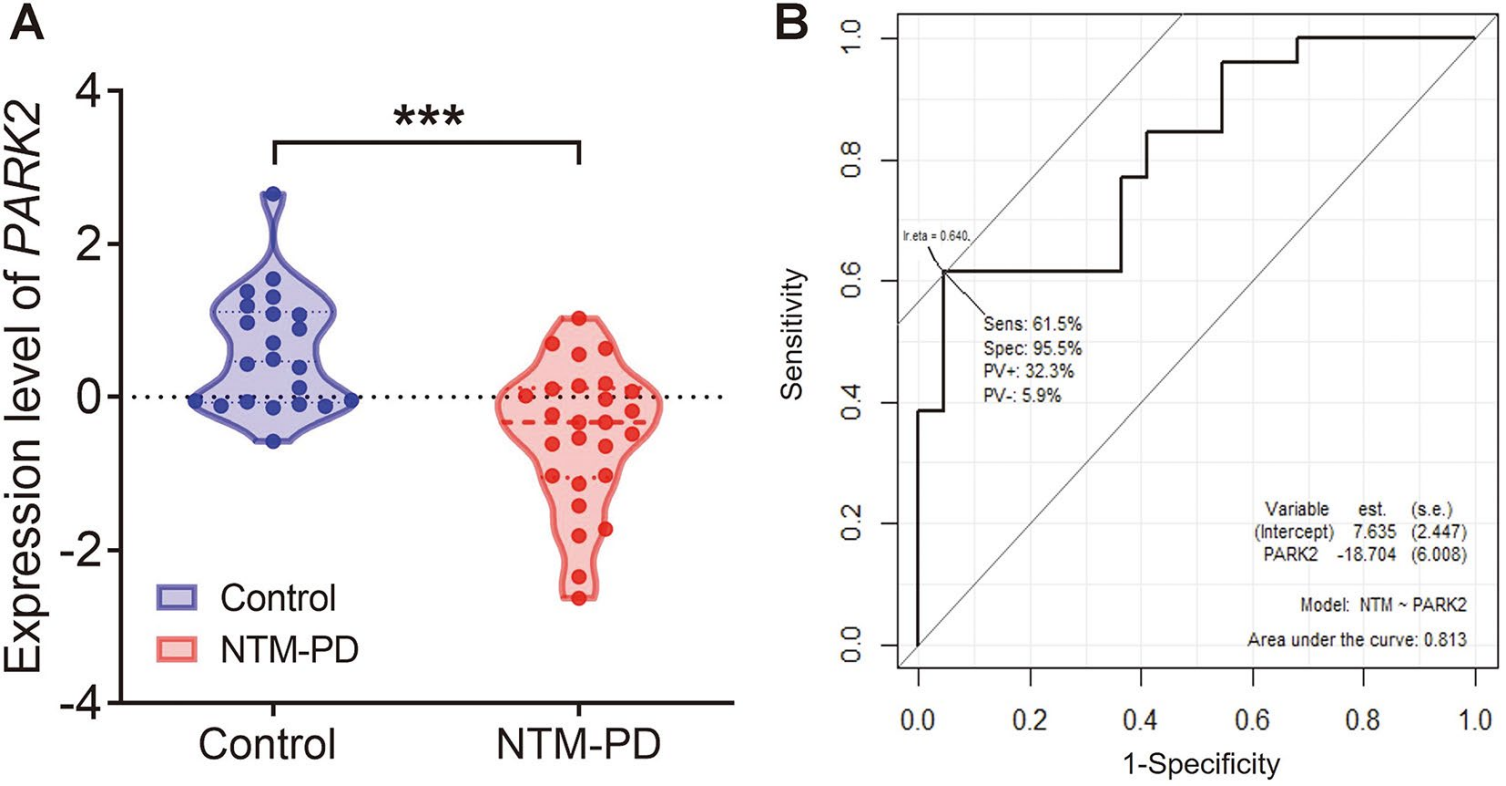
The *PARK2* gene for diagnosing NTM-PD. (**A**) Expression levels of *PARK2* in individuals with NTM-PD and healthy controls (*P* = 0.001). (**B**) The receiver operating characteristic curves depicting the predictive potential of the *PARK2* gene in classifying a sample as NTM-PD or control

The expression of *PARK2* was significantly downregulated in the NTM-PD group compared to that in the control group (Fig. 4A). However, the expression levels of *PARK2* in the pre- and post-treatment samples from patients with NTM-PD were similar (Supplementary Fig. 4). The AUC was 0.813 (95% confidence interval, 0.694–0.932), suggesting satisfactory discriminatory ability (sensitivity, 61.5%; specificity, 95.5%; positive predictive value, 32.3%; and negative predictive value, 5.9%) (Fig. 4B).

## Discussion

This study represents the whole blood RNA expression profiles of 22 healthy controls and 26 NTM-PD patients from a Korean patient cohort. We identified 65 DEGs in patients with NTM-PD compared with healthy controls. The *PARK2* gene was found to be downregulated in patients with NTM-PD, but the expression levels of *PARK2* in samples from patients before and after treatment were similar. These results indicate that the decreased levels of *PARK2* could be associated with host susceptibility rather than with a response to infection or disease severity.

Blood-based transcriptomic analysis is widely used for diagnosis of various diseases, including asthma, acute leukemia, and inflammatory bowel diseases [22–24]. In infectious diseases, diverse external stimuli cause changes in mRNA expression via transcriptional responses [25]. Direct pathogen detection is not always possible in individuals with various conditions; therefore, several studies have investigated blood-based transcriptomic signatures in infectious diseases, including tuberculosis [26]. Whole-blood genetic signatures have demonstrated promising diagnostic outcomes in assessing the risk of tuberculosis progression and in monitoring treatment response [27–29]. Moreover, a point-of-care triage test for tuberculosis using fingerstick blood achieved the minimum target product profile set by the World Health Organization (at least 90% sensitivity and 70% specificity) in the interim analysis [30]. 

Few studies have investigated genetic signatures of NTM-PDs. Matsuyama et al. [31]. conducted RNA sequencing of the NTM-infected human respiratory epithelium and found that genes related to cilia were reportedly downregulated, whereas those related to cytokines, chemokines, and cholesterol biosynthesis were upregulated. Cowman et al. [32]. performed a microarray analysis of whole-blood gene expression in 25 patients with NTM-PD and 27 controls. They reported the downregulation of 213 transcripts associated with T cell signaling, including the *IFNG* (IFN-γ) gene, which plays an essential role in antimycobacterial immunity. We applied these gene sets to our current data but no distinct clustering pattern was observed in the PCA plots (Supplementary Fig. 5). Cho et al. [33]. conducted a genome-wide association study involving 403 patients with NTM-PD and 306 healthy controls in Korea. They indicated that expression levels of the proapoptotic *STK17A* (serine/threonine kinase 17a) gene may be associated with susceptibility to NTM-PD. We also examined the expression of previously reported NTM-PD-related genes [33]. We explored the FDR and *P*-value of 44 genes from previous studies, but none showed statistical significance in distinguishing between patients with NTM-PD and healthy controls in the current data (Supplementary Table 4).

Despite these efforts, there is limited evidence regarding the genetic characteristics of NTM-PD, and the results are inconsistent. The *IFNG* gene, which was found to be downregulated in a study by Cowman et al. [32]. , was not identified as a differentially expressed gene by Cho et al. [33]. (FDR = 0.709, *P* = 0.185). The alteration in the expression of the *STK17A* gene, reported by Cho et al. [33]. , was not significant in the results of Cowman et al. [32]. In the present study, the expression levels of previously reported NTM-PD-related genes, including *IFNG* and *STK17A*, were similar between the case and control groups (Supplementary Table S4). Moreover, the gene lists from Cowman et al. [32]. could not distinguish between case and control groups in the current study (Supplementary Fig. 5). Only one gene, *DOCK9* (a dedicator of cytokinesis 9), overlapped between the current study and that by Cowman et al. [32]. 

Limited commonality among genetic signatures was also observed in studies on tuberculosis; 563 out of 721 genes were detected only once among 30 studies [27]. There are several possible explanations for the discrepancy. First, research on human cells involves diversity in the types of samples used, such as peripheral blood mononuclear cells, bronchoalveolar lavage fluid, and whole blood [34]. Second, the study population varied in age, infecting species, comorbidities, treatment stage, and geographic location. Third, the substances used to stimulate cytokine assay studies were not consistent and included phytohemagglutinin, lipopolysaccharides, and neutralized bacteria [34]. Therefore, caution is needed when comparing and interpreting the results, and further integrated analyses are required.

One of the notable findings from the current study is that PARK2 was downregulated in participants with NTM-PD. Mutations in the *PARK2* gene increase the risk of developing Parkinson’s disease [35]. However, polymorphisms in the regulatory region of *PARK2* lead to reduced expression of the PARK2 protein (Parkin), which has been linked to a higher susceptibility to intracellular pathogens such as *Mycobacterium leprae*, *Mycobacterium ulcerans*, and *Salmonella enterica* serovar Typhi [36–39].

Autophagy serves as an innate immune response that eliminates intracellular pathogens [40]. Parkin, an E3 ubiquitin ligase, plays a role in this process; Parkin-mediated ubiquitination recruits ubiquitin adaptors, promoting the autophagic targeting of mycobacteria [41]. Parkin also influences T-cell stimulation in the mitochondrial antigen presentation pathway, [42] and silencing of PARK2 reduces the generation of pyroptotic cells stimulated by inflammatory factors [43]. Moreover, the downregulation of Parkin resulted in decreased interleukin-6 and monocyte chemoattractant protein 1 (MCP-1/*CCL2*) production, suggesting that Parkin influences multiple immune-related pathways [44]. In this study, the expression levels of *PARK2* was downregulated in the NTM-PD group, but did not alter after successful treatment of NTM-PD. Therefore, decreased expression of *PARK2* may be associated with the development of NTM-PD rather than with a response to infection or disease severity. However, cautious interpretation would be required because *PARK2* is not a disease-specific genes associated with NTM-PD.

Various genes were differentially expressed between the case and control groups (Supplementary Tables S1 andS2). One of these genes, *RAB6*, encodes a small GTPase that regulates endosomal trafficking pathways and binds ligands from *Mycobacterium tuberculosis* to mucosal-associated invariant T cells in an early response to infection [45]. *RAB6A* expression was upregulated in the case group, possibly owing to NTM infection. Another upregulated gene, *P2RY12*, plays a critical role in the inflammatory response [46]. 

Certain genes have been linked to chronic obstructive pulmonary disorder (COPD), which is a common comorbidity of NTM-PD. Type IV collagen, the most abundant non-fibrillar collagen in the lungs, is associated with basement membrane integrity. Degradation of COL4A3 is associated with the disease activity of asthma and COPD [47, 48]. As extracellular matrix proteins in the lungs play a critical role in the adhesion and invasion of various pathogens, the decreased levels of *COL4A3* and *COL4A4* observed in this study may be related to NTM infection, although evidence from previous studies is insufficient [49]. *LEPR* gene polymorphisms are linked to lung function decline in COPD, [50] and *NSUN3* is associated with lung cancer development in COPD [51]. *HIVEP2* is included in a blood-based transcriptomic risk score for COPD, which is associated with lung function decline and COPD-related traits [52]. Further investigations are required to identify the roles of these genes in the pathogenesis of NTM-PD.

This study has several limitations. First, the case-control, observational, and cross-sectional designs did not allow for any conclusions regarding causality. Second, the participants were limited to those from a single center in Korea. Therefore, further studies are required to validate our findings in experimental settings involving diverse ethnic populations.

In conclusion, we identified the genetic signatures associated with NTM-PD in a cohort of Korean patients. Downregulated *PARK2* could potentially serve as a susceptibility factor. These findings improve the understanding of the clinical characteristics of NTM-PD.

### Electronic supplementary material

Below is the link to the electronic supplementary material.

Supplementary Material 1

## Data Availability

All data used or analyzed in this study are available from the corresponding author.
