# Supplementary material for: PARK2 as a susceptibility factor for nontuberculous mycobacterial pulmonary disease
Source: Respir Res. 2024 Aug 14;25:310. doi: 10.1186/s12931-024-02946-4 (PMC11325611; doi:10.1186/s12931-024-02946-4)
Supplement: Supplementary file 1 — Supplementary Material 1 [file 12931_2024_2946_MOESM1_ESM.docx]

**Supplementary Information**

**Title: *PARK2* as a susceptibility factor for nontuberculous mycobacterial pulmonary disease**

Youngmok Park^1,2, †^, Ji Won Hong^3, †^, Eunsol Ahn^4^, Heon Yung Gee^3,5, *^, Young Ae Kang^1,6, *^

^1^Division of Pulmonary and Critical Care Medicine, Department of Internal Medicine, Severance Hospital, Yonsei University College of Medicine, Seoul, Republic of Korea

^2^Institute for Innovation in Digital Healthcare, Yonsei University, Seoul, Republic of Korea

^3^Departments of Pharmacology, Graduate School of Medical Science, Brain Korea 21 Project, Yonsei University College of Medicine, Seoul, Republic of Korea

^4^Division of Vaccine Research, International Tuberculosis Research Center, Seoul, Republic of Korea

^5^Woo Choo Lee Institute for Precision Drug Development, Seoul, Republic of Korea

^6^Institute of Immunology and Immunological Diseases, Yonsei University College of Medicine, Seoul, Republic of Korea

**Supplementary Figures**


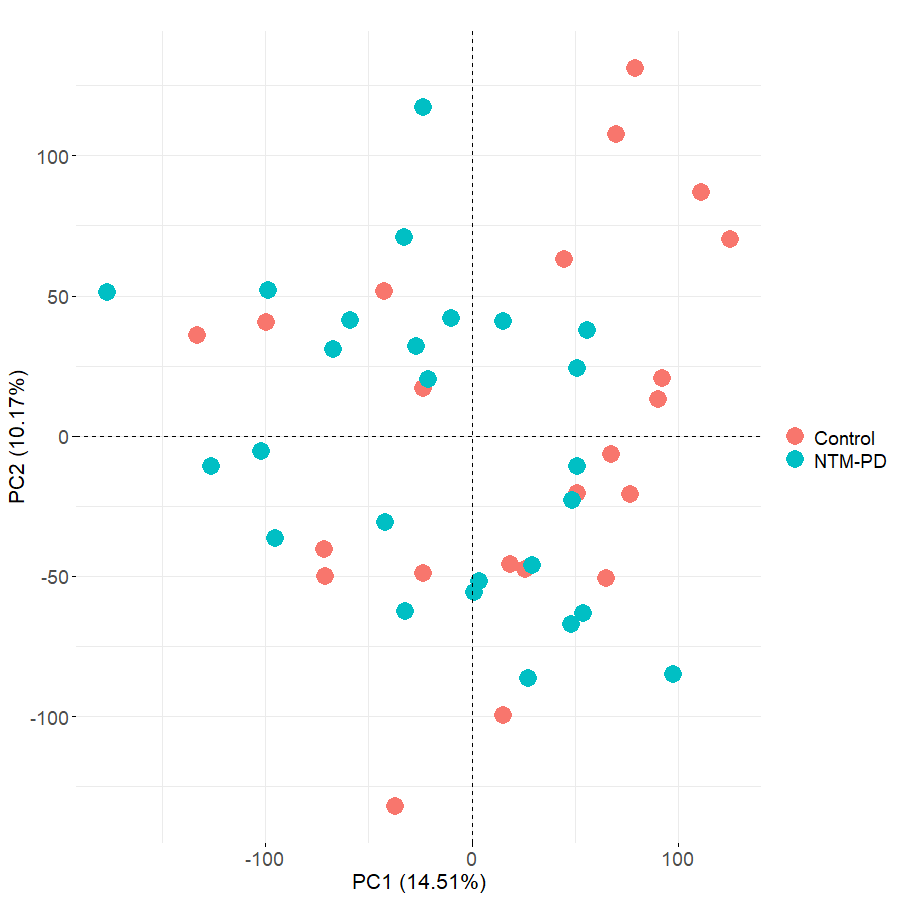


Figure S1. Principal component analysis depicting the unsupervised clustering of the case and control groups. Abbreviations: NTM-PD, nontuberculous mycobacterial pulmonary disease; PC, principal component.

Molecular function

Cellular component


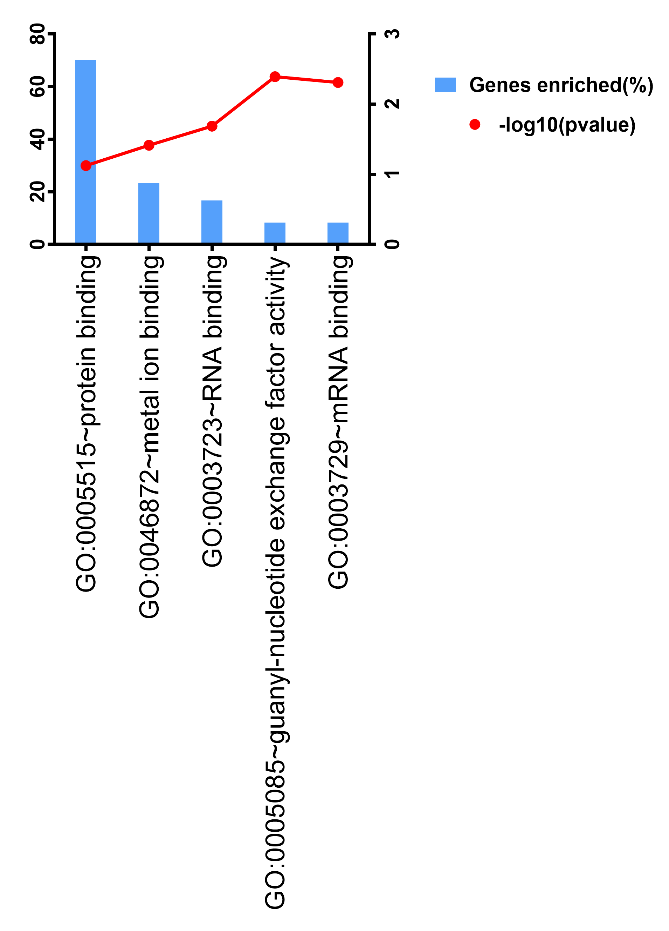


**A**

**B**


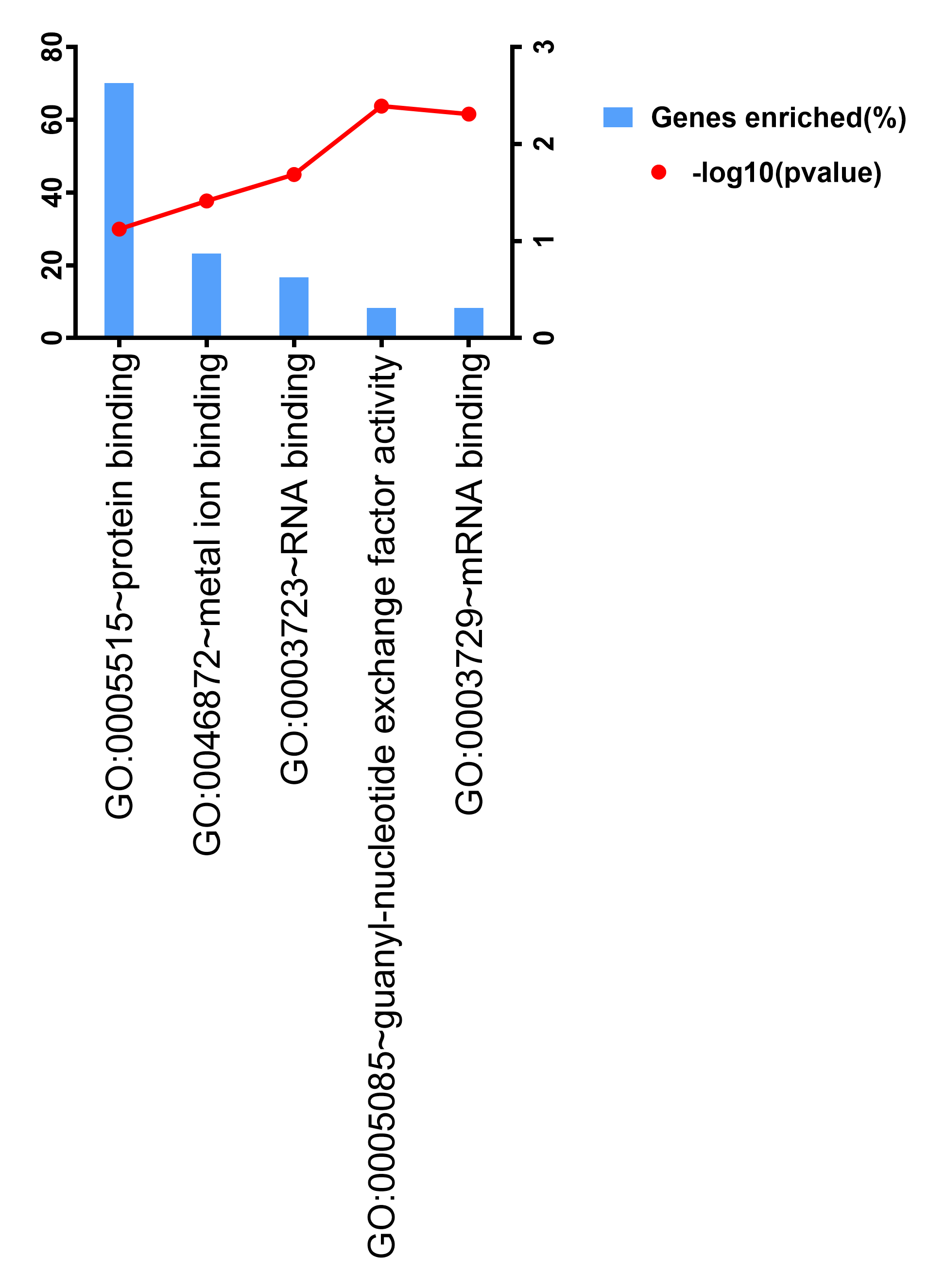

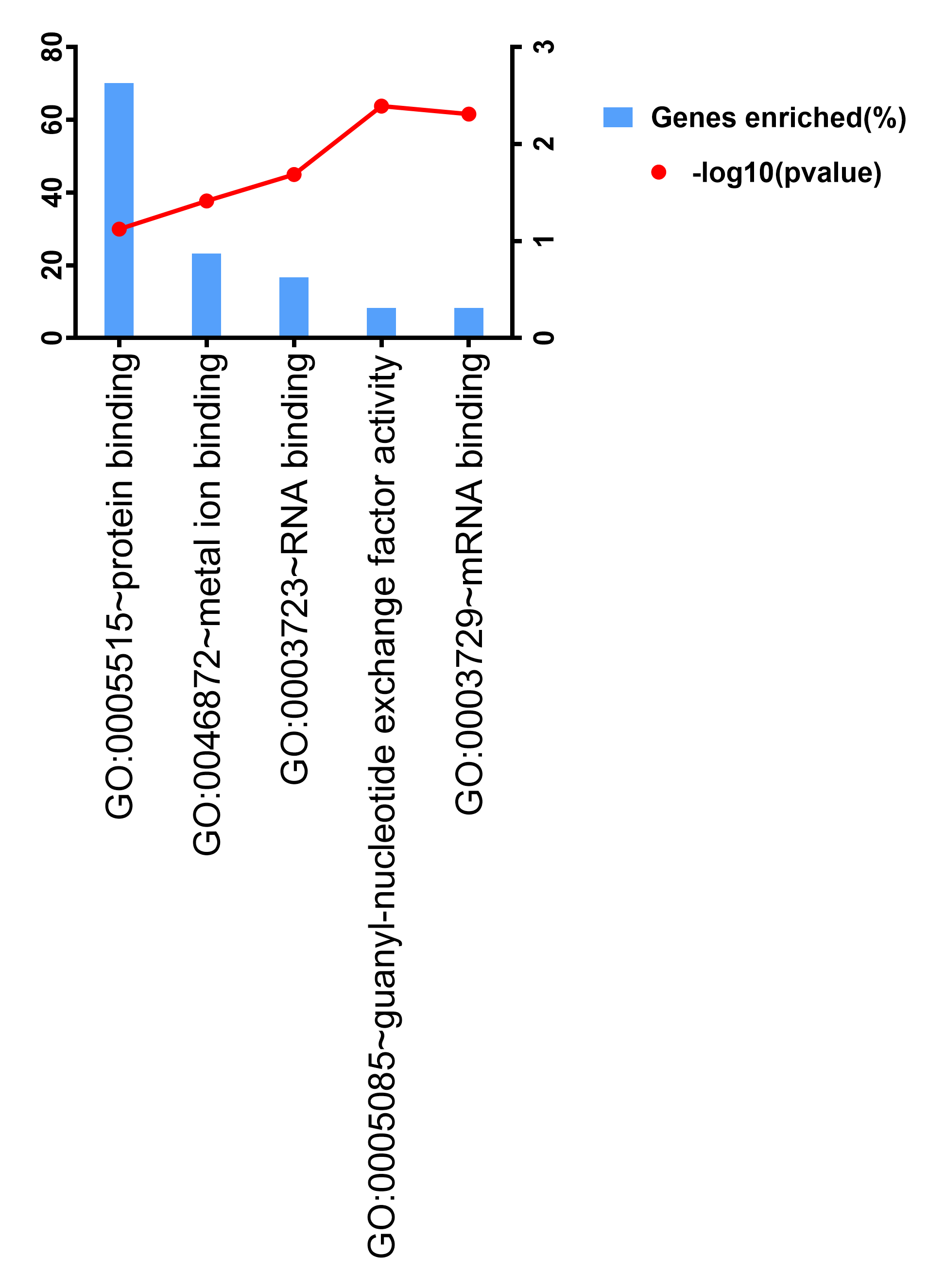

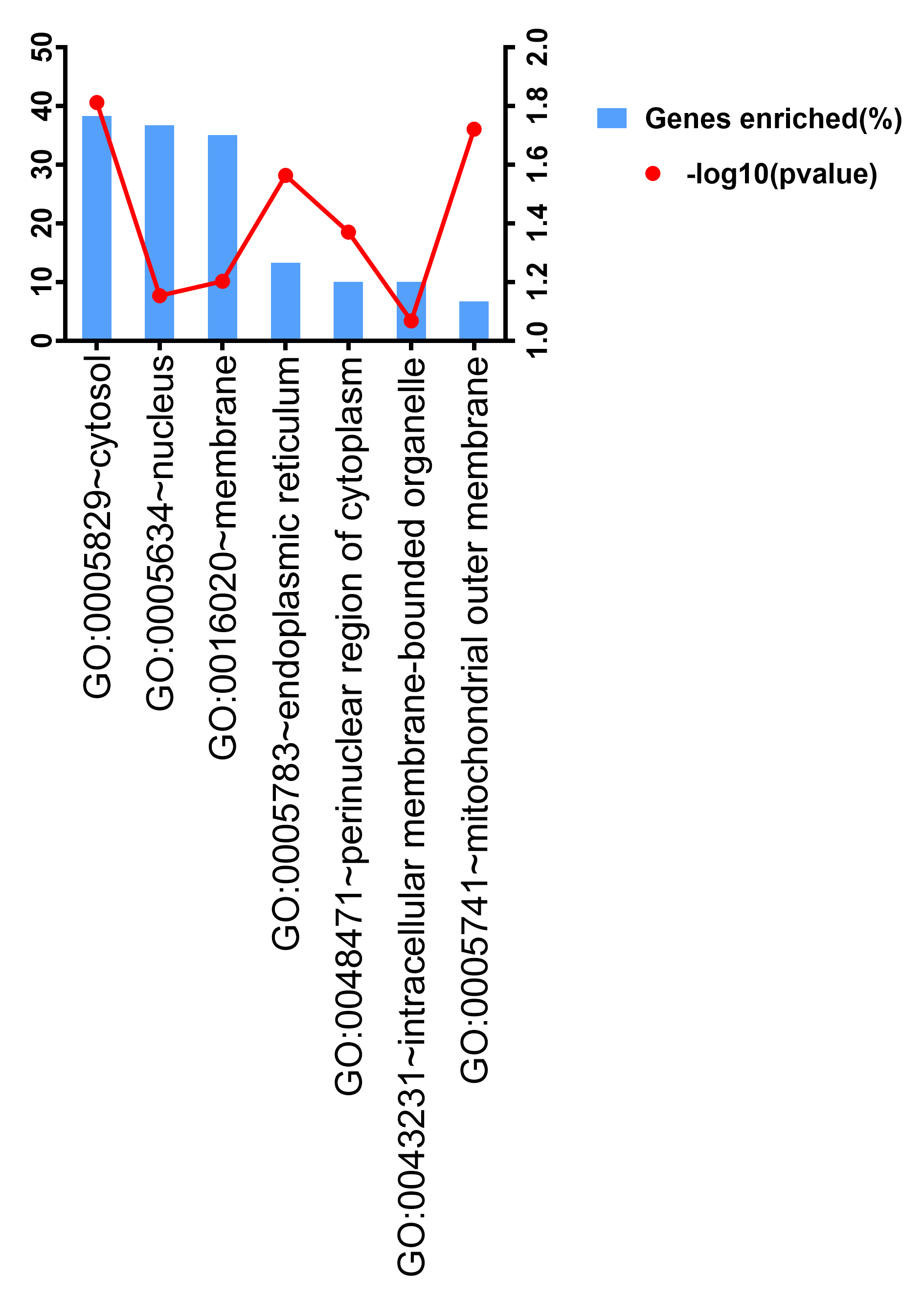


Figure S2. DAVID functional GO analysis of Differentially Expressed Genes (DEGs). (A) Molecular function, (B) Cellular component. The bars represent the gene enrichment ratio for each term, and the red lines indicate -log10 (*P*-values).


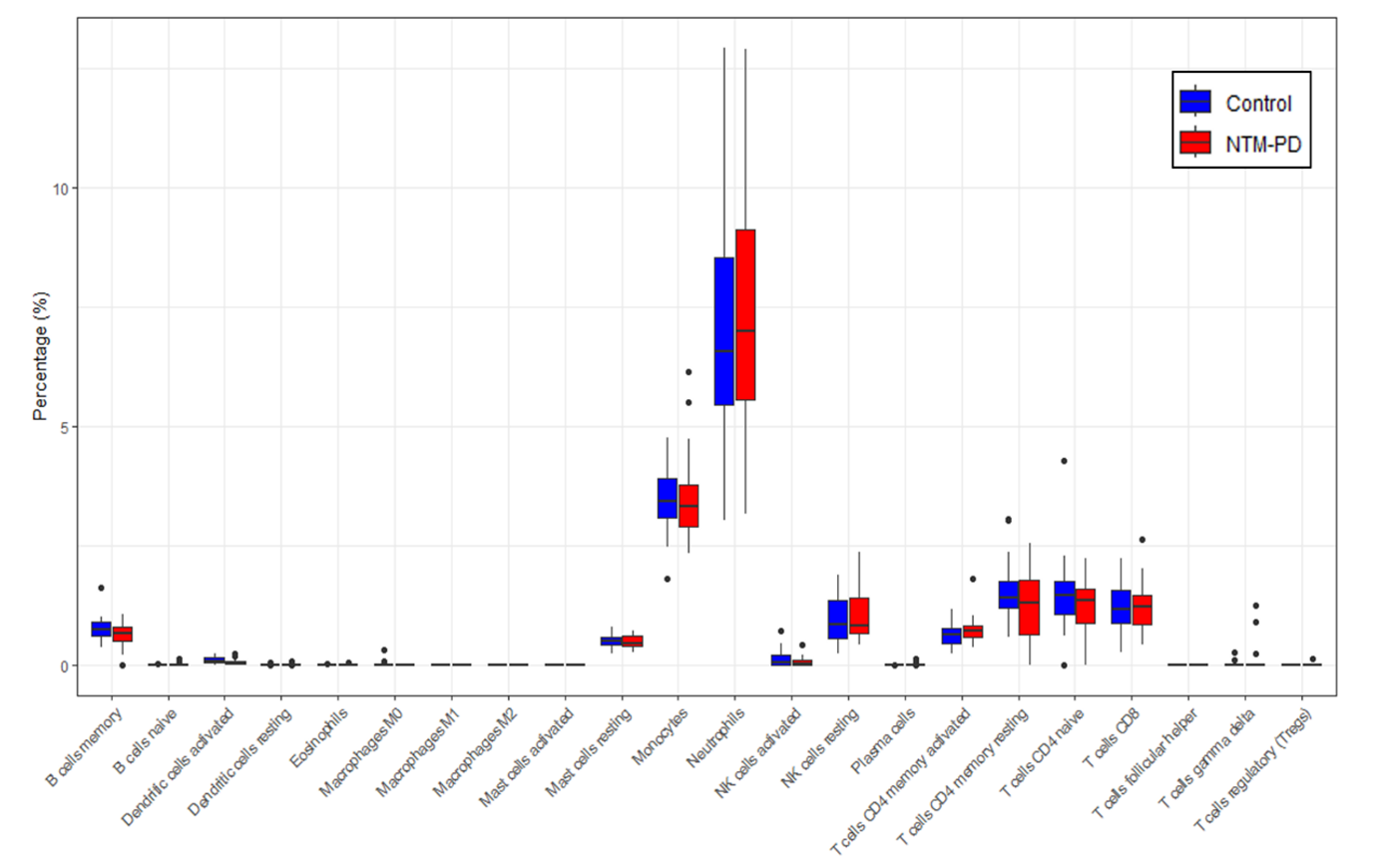


Figure S3. Proportions of 22 types of immune cells between the case and control groups.

Each box plot represents the interquartile range of the cell proportions, with the line inside the box indicating the median. The whiskers extend to 1.5 times the interquartile range. Any individual data points beyond the whiskers are considered outliers.

Abbreviations: NTM-PD, nontuberculous mycobacterial pulmonary disease.


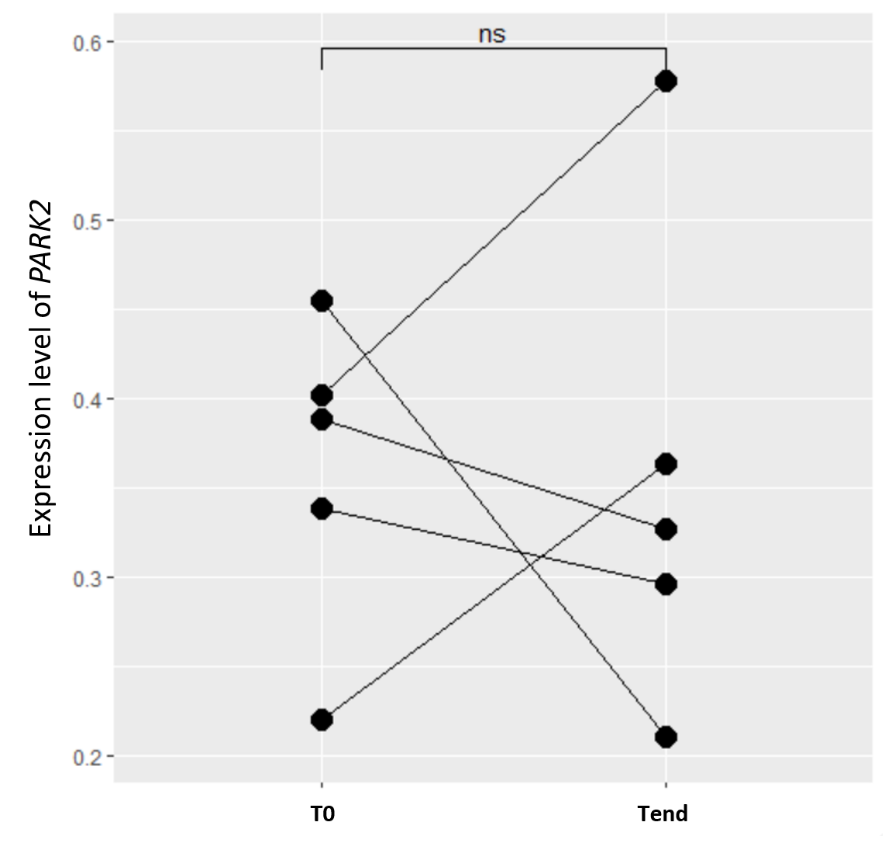


Figure S4. Expression levels of the *PARK2* gene between pre-treatment and post-treatment samples of NTM-PD. T0 indicates samples collected at the beginning of the treatment, while Tend represents samples collected at the end of the treatment. The two groups had no statistical differences (P > 0.05).


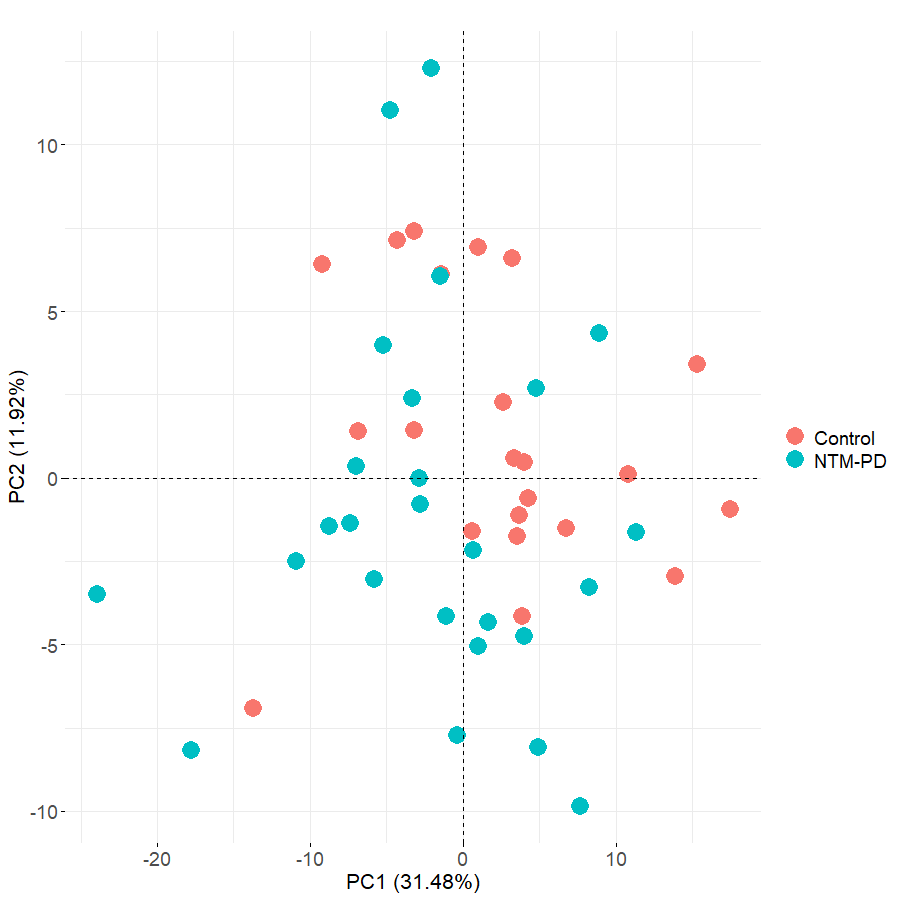


Figure S5. Principal component analysis using the Cowan *et al.* gene sets between the case and control groups. Abbreviations: NTM-PD, nontuberculous mycobacterial pulmonary disease; PC, principal component.

**Supplementary Tables**

Table S1. Upregulated genes in the case group compared with the control group

| Rank | Gene name | Description | Entrez ID | Fold change | *P* value |
| --- | --- | --- | --- | --- | --- |
| 1 | *GCSAML (C1orf150)* | germinal center associated signaling and motility like | 148823 | 1.760 | 0.020 |
| 2 | *MYCT1* | MYC target 1 | 80177 | 1.584 | 0.015 |
| 3 | *SUCNR1* | succinate receptor 1 | 56670 | 1.564 | 0.047 |
| 4 | *LEPR* | leptin receptor | 3953 | 1.376 | 0.003 |
| 5 | *CLIC2* | chloride intracellular channel 2 | 1193 | 1.305 | 0.047 |
| 6 | *P2RY12* | purinergic receptor P2Y12 | 64805 | 1.296 | 0.020 |
| 7 | *CISD2* | CDGSH iron sulfur domain 2 | 493856 | 1.262 | 0.019 |
| 8 | *CCRL2* | C-C motif chemokine receptor like 2 | 9034 | 1.255 | 0.045 |
| 9 | *C9orf40* | chromosome 9 open reading frame 40 | 55071 | 1.255 | 0.028 |
| 10 | *ARL4A* | ADP ribosylation factor like GTPase 4A | 10124 | 1.241 | 0.010 |
| 11 | *MOSPD1* | motile sperm domain containing 1 | 56180 | 1.207 | 0.006 |
| 12 | *STMP1 (C7orf73)* | short transmembrane mitochondrial protein 1 | 647087 | 1.154 | 0.045 |
| 13 | *YOD1* | YOD1 deubiquitinase | 55432 | 1.148 | 0.020 |
| 14 | *NSUN3* | NOP2/Sun RNA methyltransferase 3 | 63899 | 1.131 | 0.020 |
| 15 | *CREG1* | cellular repressor of E1A stimulated genes 1 | 8804 | 1.113 | 0.018 |
| 16 | *NT5C3 (NT5C3A)* | 5'-nucleotidase, cytosolic IIIA | 51251 | 1.113 | 0.032 |
| 17 | *YIPF6* | Yip1 domain family member 6 | 286451 | 1.089 | 0.020 |
| 18 | *BNIP3L* | BCL2 interacting protein 3 like | 665 | 1.083 | 0.046 |
| 19 | *RWDD4* | RWD domain containing 4 | 201965 | 1.078 | 0.045 |
| 20 | *STOM* | stomatin | 2040 | 1.070 | 0.047 |
| 21 | *RAB6A* | RAB6A, member RAS oncogene family | 5870 | 1.058 | 0.030 |

Table S2. Downregulated genes in the case group compared with the control group

| Rank | Gene name | Description | Entrez ID | Fold change | *P* value |
| --- | --- | --- | --- | --- | --- |
| 1 | *MYBPH* | myosin binding protein H | 4608 | –3.501 | 0.032 |
| 2 | *BFSP2* | beaded filament structural protein 2 | 8419 | –2.912 | 0.003 |
| 3 | *COL4A3* | collagen type IV alpha 3 chain | 1285 | –1.905 | 0.006 |
| 4 | *PTPRB* | protein tyrosine phosphatase receptor type B | 5787 | –1.655 | 0.047 |
| 5 | *COL4A4* | collagen type IV alpha 4 chain | 1286 | –1.529 | 0.003 |
| 6 | *HNRNPA1P70 (LOC341333)* | heterogeneous nuclear ribonucleoprotein A1 pseudogene 70 | 341333 | –1.396 | 0.027 |
| 7 | *PARK2* | parkin RBR E3 ubiquitin protein ligase | 5071 | –1.314 | 0.047 |
| 8 | *PLXNA1* | Plexin A1 | 5361 | –1.306 | 0.047 |
| 9 | *ASS1P1* | Argininosuccinate synthetase 1 pseudogene 1 | 442167 | –1.274 | 0.047 |
| 10 | *PVT1* | Pvt1 oncogene | 5820 | –1.269 | 0.010 |
| 11 | *LINC00544 (LOC440131)* | long intergenic non-protein coding RNA 544 | 440131 | –1.261 | 0.047 |
| 12 | *ZC3H12B* | zinc finger CCCH-type containing 12B | 340554 | –1.244 | 0.047 |
| 13 | *TMEM63A* | transmembrane protein 63A | 9725 | –1.170 | 0.005 |
| 14 | *TSC1* | TSC complex subunit 1 | 7248 | –1.123 | 0.047 |
| 15 | *DOCK9* | dedicator of cytokinesis 9 | 23348 | –1.116 | 0.020 |
| 16 | *CTC1* | CST telomere replication complex component 1 | 80169 | –1.116 | 0.047 |
| 17 | *DGCR8* | DGCR8 microprocessor complex subunit | 54487 | –1.115 | 0.047 |
| 18 | *PAN2* | poly(A) specific ribonuclease subunit PAN2 | 9924 | –1.115 | 0.032 |
| 19 | *SFI1* | SFI1 centrin binding protein | 9814 | –1.107 | 0.018 |
| 20 | *LUC7L* | LUC7 like | 55692 | –1.101 | 0.047 |
| 21 | *SGSM2* | small G protein signaling modulator 2 | 9905 | –1.100 | 0.047 |
| 22 | *ASXL1* | ASXL transcriptional regulator 1 | 171023 | –1.100 | 0.018 |
| 23 | *TRAF3* | TNF receptor associated factor 3 | 7187 | –1.099 | 0.036 |
| 24 | *RBM14* | RNA binding motif protein 14 | 10432 | –1.096 | 0.047 |
| 25 | *PLEC* | plectin | 5339 | –1.086 | 0.019 |
| 26 | *CHD3* | chromodomain helicase DNA binding protein 3 | 1107 | –1.085 | 0.038 |
| 27 | *ZCCHC11* | terminal uridylyl transferase 4 | 23318 | –1.081 | 0.047 |
| 28 | *HIVEP2* | HIVEP zinc finger 2 | 3097 | –1.069 | 0.020 |
| 29 | *DIDO1* | death inducer-obliterator 1 | 11083 | –1.067 | 0.005 |
| 30 | *ANKZF1* | ankyrin repeat and zinc finger peptidyl tRNA hydrolase 1 | 55139 | –1.066 | 0.028 |
| 31 | *PBXIP1* | PBX homeobox interacting protein 1 | 57326 | –1.064 | 0.032 |
| 32 | *RPL36AL* | ribosomal protein L36a like | 6166 | –1.063 | 0.032 |
| 33 | *STX16* | syntaxin 16 | 8675 | –1.063 | 0.020 |
| 34 | *SFSWAP* | splicing factor SWAP | 6433 | –1.061 | 0.031 |
| 35 | *WHSC1L1* | nuclear receptor binding SET domain protein 3 | 54903 | –1.054 | 0.027 |
| 36 | *RASGRP2* | RAS guanyl releasing protein 2 | 10235 | –1.052 | 0.032 |
| 37 | *CELF1* | CUGBP Elav-like family member 1 | 10658 | –1.052 | 0.027 |
| 38 | *ELMO1* | engulfment and cell motility 1 | 9844 | –1.051 | 0.047 |
| 39 | *FAM120A* | family with sequence similarity 120 member A | 23196 | –1.051 | 0.044 |
| 40 | *KLF6* | KLF transcription factor 6 | 1316 | –1.047 | 0.047 |
| 41 | *HMHA1* | Rho GTPase activating protein 45 | 23526 | –1.044 | 0.047 |
| 42 | *ENTPD4* | ectonucleoside triphosphate diphosphohydrolase 4 | 9583 | –1.042 | 0.018 |
| 43 | *ITSN2* | intersectin 2 | 50618 | –1.035 | 0.047 |
| 44 | *RBM5* | RNA binding motif protein 5 | 10181 | 1.033 | 0.049 |

Table S3. Modules of protein-protein interaction networks

| Cluster | Description | Score | Nodes | Edges | Node IDs |
| --- | --- | --- | --- | --- | --- |
| 1 | Mitophagy, Immune response | 3.333 | 4 | 5 | BNIP3L, CISD2, PARK2, TRAF3 |
| 2 | Regulation of GTPase activity | 3 | 3 | 3 | DOCK9, ELMO1, ITSN2 |
| 3 | Retrograde transport, endosome to Golgi, trans-Golgi network | 3 | 3 | 3 | RAB6A, STX16, YIPF6 |
| 4 | Regulation of GTPase activity (closely linked to trans-Golgi network) | 3 | 3 | 3 | ARL4A, HMHA1, RASGRP2 |

Table S4. Analysis of genes previously reported to be associated with NTM-PD in the current study samples

| Gene | FDR | *P-*value | Gene | FDR | *P-*value |
| --- | --- | --- | --- | --- | --- |
| *GATA2* | 0.188 | 0.003 | *TIGIT* | 0.860 | 0.514 |
| *LDHB* | 0.232 | 0.009 | *FLJ45825* | 0.871 | 0.530 |
| *PSPH* | 0.311 | 0.019 | *MST1R* | 0.878 | 0.540 |
| *NELL2* | 0.409 | 0.036 | *ANKRD6* | 0.885 | 0.552 |
| *SLC29A1* | 0.462 | 0.051 | *XCL1* | 0.902 | 0.578 |
| *GZMK* | 0.511 | 0.137 | *MAP2K4* | 0.919 | 0.610 |
| *IFNGR1* | 0.511 | 0.117 | *ISG15* | 0.944 | 0.653 |
| *MPEG1* | 0.511 | 0.074 | *STK17A* | 0.971 | 0.706 |
| *MUC12* | 0.511 | 0.102 | *XCL2* | 0.975 | 0.714 |
| *PPIH* | 0.511 | 0.090 | *TPBG* | 0.989 | 0.740 |
| *NFATC2* | 0.530 | 0.161 | *CFTR* | 1 | 0.818 |
| *IFNG* | 0.540 | 0.170 | *FAHD2A* | 1 | 0.862 |
| *TTK* | 0.540 | 0.171 | *IFNGR2* | 1 | 0.954 |
| *IL12RB1* | 0.598 | 0.212 | *IL2RB* | 1 | 0.917 |
| *RCOR3* | 0.652 | 0.258 | *IRF8* | 1 | 0.834 |
| *AK5* | 0.664 | 0.275 | *PMS2P1* | 1 | 0.891 |
| *KRT83* | 0.683 | 0.293 | *SAMD3* | 1 | 0.806 |
| *ORC3* | 0.704 | 0.314 | *SLC11A1* | 1 | 0.998 |
| *PZP* | 0.722 | 0.332 | *STAT1* | 1 | 0.958 |
| *CRTAM* | 0.734 | 0.344 | *TARP* | 1 | 0.989 |
| *A2M* | 0.736 | 0.347 | *GUSBP14* | NA | NA |
| *FCRL3* | 0.841 | 0.488 | *IFNLR1* | NA | NA |

NA, not available.
